# The need for and acceptability of a curriculum to train nursing and medical students in the sexual healthcare of clients with female genital mutilation/cutting in Tanzania

**DOI:** 10.1186/s12905-024-03034-x

**Published:** 2024-03-26

**Authors:** Dorkasi L. Mwakawanga, Agnes F. Massae, Nidhi Kohli, Gift Gadiel Lukumay, Corissa T. Rohloff, Stella Emmanuel Mushy, Lucy R. Mgopa, Dickson Ally Mkoka, Ever Mkonyi, Maria Trent, Michael W. Ross, B. R. Simon Rosser, Jennifer Connor

**Affiliations:** 1 Muhimbili University of Health and Allied Sciences, United Nations Rd, Dar es Salaam, Tanzania; 2 University of Minnesota, #300, 1300 S. 2nd St., Minneapolis, MN 55454 USA; 3 Johns Hopkins University, 200 N. Wolfe Street, Baltimore, MD 21287 USA

**Keywords:** Female genital cutting, Mutilation, Sexual health, Curriculum, Healthcare professionals, Tanzania

## Abstract

**Background:**

Female genital mutilation/cutting (FGM/C) is tied to one of the most conservative cultures in the Mediterranean and Sub-Saharan Africa. More than 200 million girls and women in 30 African, Asian and the middle Eastern countries have undergone FGM/C. However, healthcare professionals are not adequately trained to prevent and manage FGM/C-related complications including sexual health problems. This study aimed to assess the need and acceptability of a curriculum to train nursing and medical students in the sexual healthcare of clients with FGM/C in Tanzania.

**Methods:**

We used a descriptive and cross sectional study design to collect and analyse information from 271 medical and 137 nursing students in Tanzania. A Qualtrics online survey was used to obtain quantitative data on training interest, previous training received, and the curriculum delivery method. Open-ended questions were used to explore their insights on significance to obtain the necessary competencies to treat and prevent FGM/C. Descriptive statistics were used to analyze quantitative data while qualitative data were analyzed using a thematic approach.

**Results:**

Almost half of the participants reported they had little to no training in sexual healthcare for women with FGM/C (47%). In all, 82.4% reported the training to be acceptable. Following thematic analysis of open-ended questions, participants expressed a desire to improve their competencies to meet the current and future sexual and psychological health needs of women and girls who have undergone FGM/C.

**Conclusion:**

It is a necessary and acceptable to develop a curriculum to train healthcare students to diagnose, treat and prevent sexual health complications related to FGM/C. In our study, designing a culturally sensitive curriculum and its delivery method, that includes practical sessions with simulated patients, was considered the most beneficial and favorable.

## Background

Female genital mutilation/cutting (FGM/C) is a major global health issue affecting girls’ and women’s quality of life [1]. More than 200 million girls and women in 30 African, Asian and the middle Eastern countries have undergone FGM/C [1, 2]. Female genital mutilation/cutting prevalence rates among women and girls aged 15–49 are highest in Somalia (98%), Guinea (97%), Djibouti (93%) [3], Gambia (56%), Mauritania (54%), and Indonesia (~ 50%) [2]. Cases of the practice have been documented from the United States, Canada, and Australia, as well as specific ethnic groups in Central and South America, as a result of international migration [2, 4–6]. In Tanzania, the estimated prevalence is 10%, with the highest rates in northern (58%) and central (47%) zones. This represents a significant reduction from 1996’s rate of 17.9% when the practice became illegal [7, 8]. However, a recent study of 324 women of reproductive age living in a high prevalence region within Tanzania, revealed that 81.8% had undergone FGM/C and 68.8% were willing to practice genital cutting [9]. Another Tanzanian regional study found that 69.2% of 675 women self-reported FGM/C; yet records at the regional hospital indicated that over 96% of women who gave birth during the same time period had experienced FGM/C [10]. This discrepancy led the researchers to believe that the practice has become increasingly hidden due to legal concerns and possible stigma. Another implication is that healthcare providers, in particular those in maternal health fields, have an opportunity to engage patients in discussion about FGM/C.

FGM/C is defined as any procedure that involves the partial or total removal of external genitalia or other injury to the female genital organs for non-medical reasons [2]. The World Health Organization [2, 11] classifies FGM/C into 4 types; Type I clitoridectomy is the partial or total removal of the clitoris. Type II excision refers to partial or total removal of the clitoris together with partial or total excision of the labia minora. Type III infibulation is a partial or total removal of the external genitalia and stitching of the vaginal opening. Type IV involves other traditional genital procedures such as pricking, stretching the clitoris and/or surrounding tissues, piercing and cauterizing. Depending on the type and traditions of a community, FGM/C is performed at different ages, including during the first weeks of life, childhood, adolescence or before pregnancy [12].

FGM/C has no known health benefits. On the contrary, it subjects girls and women to serious health consequences throughout their lives [13]. The short-term effects include injuries to adjacent tissues, bleeding, severe pain, infections and even death [14]. The long-term consequences include chronic pain, recurring urinary tract infections, post-traumatic stress disorder, menstrual problems, bacterial vaginosis [15], and decreased sexual enjoyment often due to pain [16]. Moreover, FGM/C has resulted in obstetrical complications such as prolonged labour, obstetrical tears, increased risk of caesarean section, postpartum haemorrhage, maternal mortality, neonatal deaths, still births and low birth weight [17, 18]. According to a WHO report, an annual expenditure of at least US$ 1.4 billion is incurred to treat health complications of FGM/C [19] and at least $3.7 million to treat obstetrics complications resulting from FGM/C in 6 African countries [20]. In light of this, healthcare professionals (HCPs) should be prepared to discuss and treat medical and sexual health problems for patients who have experienced FGM/C.

Several interventions, including outlawing the practice through policy, large-scale marketing campaigns to change public perceptions, education programs, community mobilization and economic empowerment programming have been implemented to eradicate FGM/C [21]. These interventions aim to modify attitudes and norms that support violence against women and girls. Regional and global organizations, such as World Health Organization (WHO), United Nations Children’s Fund (UNICEF), United Nations Population Fund (UNFPA), United Nations and African Union, have prioritized the eradication of FGM/C [22, 23]. In addition, the Sustainable Development Goal 5 (SDG 5) has an aim to eliminate FGM/C to achieve gender equality and empowerment of all women and girls by 2030 [24]. As a result of these efforts, FGM/C is gradually declining, reflecting generational trends [25]. However, high population growth in countries where the practice is prevalent suggests that the number of affected girls and women would likely increase by 2030 [25]. Additionally, there is emerging evidence that efforts to halt the practice have been hindered by other emergencies, in particular the COVID-19 pandemic and climate change disasters [26]. Unfortunately, many HCPs in regions with high FGM/C prevalence are often unaware of the many negative health consequences of FGM/C, and many lack the required training to recognize and treat them properly [2, 27, 28].

A common definition of high prevalence region is one with higher than 10% of the population reporting FGM/C [29]. As noted above, Tanzania falls just short of that definition even though there are several regions of Tanzania with possible rates as high as 96%. Despite the fact that one in ten women have experienced FGM/C, most of the resources and research by global organizations have been funneled to countries with national rates higher than 10%. Additionally, the FGM/C procedure in Tanzania is traditional and is deeply rooted in cultural and ethnic identity [9, 10], and is considered as a significant rite of passage into womanhood [7]. In many communities, it has been used to control women’s sexuality, to preserve virginity and chastity required for marriage, and to protect the honor of the community [30]. The rite confirms honor, value, identity, pride and a sense of belonging to the cultural and social group [31]. In some communities, there is a belief that religion requires FGM/C [32], yet that is not the case in Tanzania. The variability in the socio-cultural motivations to continue the practice point to the need for culturally sensitive interventions to eradicate the practice and shape the healthcare landscape.

Several studies have shown that HCPs have little knowledge and experience of FGM/C related health problems, making them uncomfortable to serve these patients, including in both high FGM/C prevalence countries and countries who serve as migration destinations [27, 33, 34]. Significant gaps have also been observed in the provision of proper and safe prenatal and childbirth care for women who have undergone FGM/C [35]. Training and provision of guidance to HCPs on FGM/C is critical and has been demonstrated to be effective in improving clinical practice and increasing advocacy efforts to eradicate the practice, yet is underutilized and sometimes discouraged in areas where FGM/C is practiced [36–40]. A possible reason for this underutilization is the perception that the topic is too sensitive [36]. Globally, there is a need for FGM/C specific training for frontline clinical practitioners such as midwives, nurses and physicians to improve their capacity to make correct diagnoses, document, report, treat, and provide psychosexual care [41–44]. Our previous research has demonstrated that tailoring sexual health training modules or coursework to a Tanzanian setting is an effective strategy for strengthening the understanding, knowledge, and skills of healthcare professionals [45]. However, no research has evaluated the need and acceptability of FGM/C training within the Tanzanian context. This paper describes survey data on the need and acceptability of a curriculum to train nursing and medical students in the sexual health care of patients with FGM/C in Tanzania.

## Methods

### Study design and setting

We conducted a descriptive and cross sectional study to answer our research questions. The study was conducted at the Muhimbili University of Health and Allied Sciences (MUHAS) in Dar es Salaam, Tanzania [45]. MUHAS trains a large population of future health professionals in Tanzania and is among the best medical universities in the African region in conducting key research.

The study was approved by the University of Minnesota Institutional Review Board (protocol number STUDY00006904), the institutional review board of MUHAS (study number DA.282/298/01.C and the [Tanzanian] National Institute for Medical Research (protocol number NIMR/HQ/R.8a/Vol.IX/3020). Given the study occurred in 2021 during the COVID pandemic, all participant procedures were conducted with a COVID prevention protocol as approved by the University of Minnesota Office of Vice President for Research.

### Participants and sample size

We used a convenience sample consisting of students who had participated in a 4-day optional training about sexual health at MUHAS. This training was delivered and evaluated through a NIH funded randomized control trial [45]. A total of 412 students were recruited through flyers on campus noticeboards and announcements in class to participate in the training. In order to recruit students with some clinical experience, medical students were recruited in their 3^rd^ or 4^th^ year and nursing and midwifery students were recruited in their 2^nd^ or 3^rd^ year. In the current manuscript, we report findings from participants who took part in a follow-up survey. Four of the baseline 412 participants did not complete the follow-up survey, resulting in a sample of 408 students (271 medical students and 137 nursing students).

### Data collection procedures

The researchers consented participants prior to data collection, including an explanation of the purpose of the survey, confidentiality, and their right to withdraw at any point. The data reported here were collected in August and December 2021. All participants completed a Qualtrics baseline survey (with sociodemographic questions) prior to participation in the RCT, and a follow-up survey three to six months later (time varied based on when they participated in the training). The questionnaires were in English and administered on the university premises.

#### Survey materials

The baseline survey included sociodemographic questions, such as gender, academic discipline, religious background, and age. The follow-up survey was designed to evaluate the sexual health training from the RCT, and identify further needs not covered in the existing training such as the health care provision of patients with FGM/C. Four fixed choice questions inquired about the participants’ (1) perspective about the acceptability of this course material through a 5 point likert scale item, (2) interest in the topic through a 5 point likert scale item, (3) preferred course delivery method (e.g., as a seminar), and (4) level of previous exposure to FGM/C education. Participants were allowed to determine if they were well educated, or had a lot, some, a little, or no previous training based on their own definition. Open-ended questions were used to collect participants’ perspectives on the importance of receiving FGM/C training, and how they would like to receive the training. Students were also asked to reflect and write down their thoughts about the importance of FGM/C training in Tanzania. Given the lack of information about this topic in Tanzania healthcare education, an open-ended question was used to elicit attitudes and experiences from students without predetermined response options [46].

### Data analysis

Quantitative data were analysed in RStudio software version 4.2.2. Due to the small number of midwifery students (N = 13) during analysis we combined them with nursing students to form a category of nursing students. Descriptive statistics were utilized to describe frequency of interest and acceptability of receiving training in sexual health care of women who have undergone FGM/C.

All the responses from open-ended questions were extracted and analysed using a thematic approach. Two researchers (DLM and JC) used line to line open coding for all responses. The 2 coders discussed discrepancies between their codes until they reached agreements. After the codes were defined inductively from the responses, the 2 researchers coded all responses. The last author reviewed the codes. Codes were examined for similarities and differences and grouped into sub-themes. The focus was on broad patterns in the data and the coded data were combined based on their relationships to form sub-themes and themes [47]. The identified themes were finalized by review and discussion with the team of researchers.

### Rigor and reliability

The methods to recruit and collect data from participants were rigorous. Though a convenience sample, the participants were recruited as part of the RCT. Extensive work went into identifying which types of students should be included in training future healthcare providers on sexual healthcare in Tanzania via key informant and focus group interviews with stakeholders prior to recruitment. These interviews were conducted with community leaders [48], experienced healthcare providers, and healthcare students [49] in Tanzania; the findings indicated that training related to general sexual healthcare should include future physicians, nurses, and midwives. MUHAS trains the highest number of healthcare providers in Tanzania, and therefore is an appropriate recruitment site. Carefully crafted research protocols allowed us to recruit a sizeable number of participants and retain them throughout the larger study, with only 1% lost to follow-up.

This manuscript adds to the field by examining an area left unexamined in Tanzania. We relied on triangulation of types of data and multiple perspectives to develop an understanding of what the training needs are [50]. Participants represented 3 disciplines. The open-text question added to the understanding of the likert scale questions. For the analysis of the open-text question, the initial coding team consisted of a nurse educator situated in Tanzania and a United States (U.S.) based academic who has extensive experience in coding qualitative data about FGM/C. Themes were reviewed and discussed with a team representing multiple disciplines (Medicine, Nursing, Midwifery, Psychology, Public Health, Family Therapy, and Sexology), and who are situated in Tanzanian and U.S. academic institutions. By utilizing a team-based approach, the data and findings were viewed through multiple perspectives and strengthen trustworthiness of the interpretation [51].

## Results

### Socio-demographic characteristics

In the sample of 408 students, 271(66.4%) were medical students and 137(33.6%) were nursing students. Participants had a mean age of 23.9 (SD = 2.3) among medical students and 24.1 (SD = 2.8) among nursing students. Two hundred (49%) participants were in their final year, while two hundred and eight (51%) participants were in their penultimate year. Moreover, 178(65.4%) of 271 students in the medicine discipline and 94(68.6%) of 137 students in the nursing discipline were male. Around 74% of participants identified as heterosexual and 93.6% of the participants were single. In all disciplines, the majority of participants were Christian 84.8%, and moderately religious 60% (see Table 1).

**Table 1:** Participants demographic characteristics

Variable	Medicine (***N*** = 271)		Nursing (***N*** = 137)	
	*n*	*%*	*n*	*%*
**Year of Study**				
Final	138	50.9	62	45.3
Penultimate	133	49.1	75	54.7
**Gender**				
Female	88	32.5	38	27.7
Male	178	66	94	68.6
Other/Prefer Not to Answer	5	1.5	5	3.6
** Age (M, SD)**	23.9	2.3	24.1	2.8
**Relationship Status**				
Single	257	94.8	125	91.2
Married (monogamously)	7	2.6	9	6.6
Cohabitating	5	1.8	2	1.5
Other	2	0.8	1	0.7
**Sexual Orientation**				
Heterosexual	224	82.6	78	56.9
Asexual	17	6.3	31	22.6
Bisexual	13	4.8	11	8
Transgender	1	0.4	4	2.9
Unsure	7	2.6	9	6.6
Prefer Not to Answer	9	3.3	4	2.9
**Religious Affiliation**				
Christian	226	83.4	120	87.6
Muslim	42	15.5	16	11.7
Other/Prefer Not to Answer	3	1.1	1	0.7
**Religiosity**				
Not at all religious	8	2.9	13	9.5
Slightly religious	59	21.7	50	36.5
Moderately religious	174	64.2	71	51.8
Very religious	15	5.5	2	1.5
Extremely religious	3	1.1	0	0
Prefer Not to Answer	12	4.4	1	0.7

### Interest and acceptability of training

Participants reported a high level of interest in receiving training on sexual healthcare of women who have experienced FGM/C. In all, 336(82.4%) participants indicated they were “very interested”, and 63(15.4%) indicated “interested”. Only 2 out of the 408 participants (0.5%) stated they were “uninterested”, and both were medical students. An additional 7(1.7%) provided a neutral answer. Almost all participants reported that this type of training is acceptable, with only 3 individuals indicating disagreement (2 medical students and 1 nursing student).

### Previous FGM/C training

**Fig. 1:**
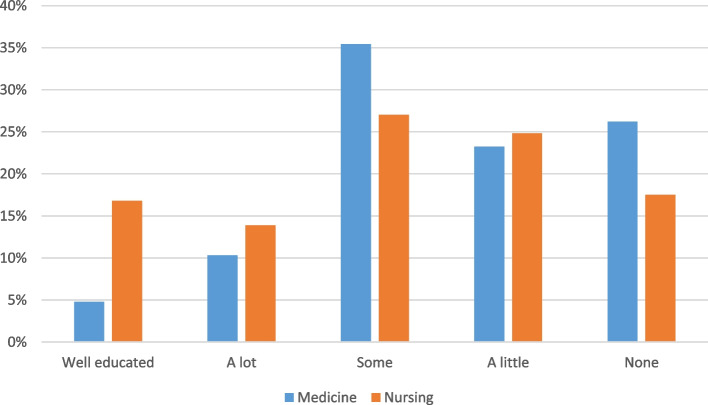
Previous training by discipline

Participants reported a range of experience in receiving previous training, as indicated in Fig. 1. Most participants did not have a high level of training in sexual healthcare of women with FGM/C – in particular within the medical discipline. Only 16.8% of nursing students and 4.8% of medical students considered themselves well educated.

### Method of curriculum delivery

**Fig. 2:**
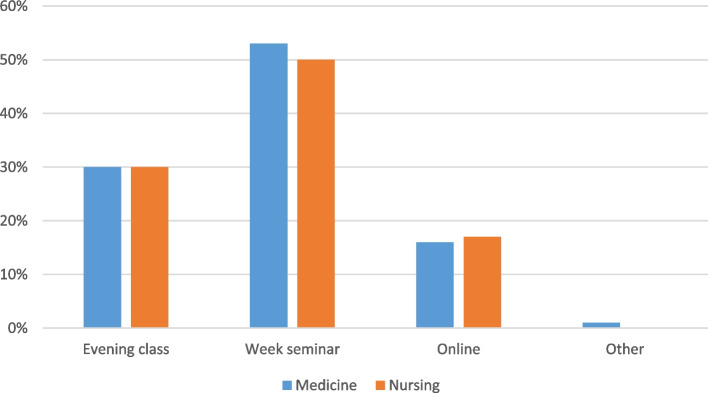
Method of curriculum delivery

Medical and nursing students answered similarly to the method of curriculum delivery they preferred, as demonstrated in Fig. 2. The results indicate a preference for in person training, with about half preferring to have a weeklong seminar held during a typical semester break.

Table 2 shows some differences identified when we compared responses between male and female participants regarding the need for and acceptability of the FGM/C curriculum training for medical and nursing students. Of all male participants, 75% strongly agreed that the curriculum was acceptable, whereas 83% of female participants reported that it would be acceptable for them to receive a curriculum. Most (86.5%) of females and (80%) males were very interested in receiving training on healthcare of women who have undergone FGM/C, with two males reporting to be uninterested to receive the training. Because so few participants indicated a lack of acceptability and interest, we were unable to test for statistical difference between groups.

**Table 2:** Need for and acceptability of FGM/C training between male and female students

Items	Male N (%)	Female N (%)
**Interest in receiving training**		
Very interested	219 (80.5)	109 (86.5)
Interested	45 (16.5)	16 (12.7)
Neutral	6 (2.2)	1 (0.8)
Uninterested	2 (0.7)	0 (0)
Very uninterested	0 (0)	0 (0)
**Level of training received before**		
Well educated	29 (10.7)	5 (4)
A lot	28 (10.3)	17 (13.5)
Some	88 (32.3)	44 (34.9)
A little	68 (25)	28 (22.2)
None	59 (21.7)	32 (25.4)
**Agreement on the acceptability of the curriculum**		
Strongly agree	206 (75.7)	105 (83.3)
Agree	59 (21.7)	19 (15.1)
Neither agree nor disagree	5 (1.8)	0 (0)
Disagree	1 (0.4)	1 (0.8)
Strongly disagree	1 (0.4)	1 (0.8)

### Thematic analysis

Three themes were identified through the analysis of the open-ended question (1) education to improve competence and confidence, (2) the role and benefits of the training on the impact of FGM/C, and (3) content and approaches for delivering the training. Table 3 shows the summary of qualitative findings, including the codes that were developed into subthemes and finally overarching themes. Notably, there were no codes or themes that indicate any concerns with FGM/C training. Though participants were asked about sexual healthcare, they spoke more broadly to include other types of healthcare such as maternal health and psychological well-being -- as will be apparent in the below quotes.

**Table 3:** Selected codes, sub-themes and themes

Selected codes	Sub-themes	Themes
Not knowing much about FGM Heard about the practice Not knowing what to do with FGM patients	Inadequate knowledge among HCPs	Education to improve the competence and confidence
Less confidence to face FGM patients Being unable to help a victim	Lack of confidence among HCPs	
Interested on part of history taking Applicability in the society Highly relevant to the society	Readiness of HCPs to be trained	
Skills is useful in effort to combat FGM is still ongoing in Tanzania Support of HCPs in addressing the issue Collective efforts to combat FGM	A desire for ending the FGM/C practice	
FGM result in emotional and psychological issues FGM leads to sexual health challenges FGM is against human rights FGM result in many health impacts	Knowledge on the FGM/C practice and its impacts	Role and benefits of training on the impact of FGM/C
Building confidence and competence Providing appropriate care Improve ability to treat FGM patients	Perceived benefits of the training to the HCPs	
Reduce childbirth complications Maintain women sexual health Protection of reproductive health Reduction of sexual violence	Perceived benefits of the training to women	
Community empowerment Reduce burden of infections Reduction of stigmatization	Perceived benefits of the training to the community	
Involving more affected areas Centering a training to vulnerable Focus on pastoralists Raising community awareness first	Adequacy involvement of the community	Content and approaches for delivering the training
Evidence why FGM is harmful Focus on psychological and emotional support How to care the related complications	Focus of the content	
Training the majority of HCPs Having more time of the training Using standardized patients	Teaching modalities	

### Education to improve the competence and confidence in dealing with FGM/C cases

Participants indicated that training is necessary for improving the competence of healthcare professionals to manage FGM/C related cases. Participants stated they feel a need for more knowledge to better serve patients. For example, many stated they heard of the practice but do not know much about it and have less ability to treat these patients. One participant was quoted saying “*With this training I would be able to help in case I encounter patients, friends and family members who outright experience FGM, and if so, offer them directive care for which will be helpful to them.” (Medical student).*

Other participants reported lacking confidence when faced with FGM/C patients and hence being unable to help a victim. For example, a medical student stated, *“I have seen some women who are victims of FGM, too bad I did not know how to go through, I hope the training would give me the confidence to attend such clients.” (Medical student).*

To improve HCPs competence and confidence in handling FGM/C patients, it is also crucial that they are ready for training. The majority of participants stated that training is essential since it is very relevant to society and its applicability will increase as this practice continues in some societies. In addition, they showed interest in learning how to incorporate patient FGM/C information while taking a sexual and medical history. One participant said, “*I would like to participate in this training, because it is highly relevant in our society” (Medical student).*

Other participants reported *“I will be much interested in learning how to take the medical history of a woman with female genital cut.” (Nursing student), “It’s quite important because it’s a major cultural practice in remote areas of our settings.” (Medical student).*

Participants demonstrated a desire to join efforts in ending the FGM/C practice. They acknowledged the existence of the practice in society and the need for skills in preventing further impacts as well as a collective effort to combat it. The participants added that the help and support from healthcare professionals is very crucial in addressing the issue. For example, a participant said, *“Skills will help in the effort to combat this practice in societies and also help the affected individuals.” (Medical student), and another similarly stated, “This will also contribute to reducing the events of FGM in Tanzanian, societies of Tanzania, particularly in the rural regions.” (Medical student).*

Another participant expressed the need of having adequate knowledge for eradication by saying, *“We need knowledge to know how we can combat and eradicate female genital cutting in areas where the community have not been educated on disadvantage of female genital cutting” (Medical student).*

### Role of training on addressing the impact of FGM/C

Participants demonstrated some comprehension of the FGM/C practice and its consequences. They stated that FGM/C is a taboo subject that violates human rights and has resulted in many health impacts, including sexual challenges. Participants asserted that FGM/C can result in emotional and psychological problems and has contributed to marriage instability as a result of sexual health challenges. *“Female genital cutting has many negative impacts like tears and excessive bleeding during delivery, which can lead to death, painful sexual intercourse, psychological trauma, and failure to enjoy sex or achieve orgasm. So, the training will help us to gain knowledge and raise community awareness about the effects of this harmful cultural practice as well as help the females who are at risk” (Medical student).*

Participants believed that the training would equip them with knowledge necessary to effectively handle FGM/C cases, hence improving their practices. For example, 1 participant remarked that, *“It will help me in identifying the sexual heath challenges experienced by women who have undergone female genital cutting and provide the necessary care” (Medical student).* In addition, they stated that training would remind HCPs of their role to provide health education to women and to protect their reproductive and sexual health, hence reducing sexual health challenges and enhancing their mental health.

Participants expressed that training HCPs in the health care needs for women with FGM/C would improve the provision of proper childbirth care for these women, hence reducing childbirth complications and saving their lives. *“It will help to reduce childbirth complications particularly postpartum haemorrhage, for this specific group, HCPs will be able to care for them and provide them with information about sexual and reproductive health” (Nursing student).*

The community benefits of the training were also emphasized, as the training will provide HCPs knowledge needed to educate society about the impacts of FGM/C. For example, HCPs will be able to raise awareness of the effects of FGM/C, minimize the burden of FGM/C related impacts such as infections and sexually transmitted diseases, and eliminate stigma. *“It is important to get trained in sexual healthcare for women in regard to FGM because we need to gain a great deal of knowledge about it in order to educate our society/community about the bad impacts of FGM” (Medical student).*

### Content and approaches for delivering an impactful training on FGM/C

Participants also urged on an emphasis on the training’s content being culturally sensitive. They further noted that it would be beneficial if the training curriculum included evidence-based explanations of why FGM/C is a detrimental practice by focusing on its impacts. In addition, they emphasized the importance of focusing on psychological and emotional assistance as well as how to treat other complications.

*“The training has to provide the facts as to why female genital mutilation is harmful, so that clinicians could know how to deal with FGM cases and properly educate the public the consequences”. (Medical student)*

The majority of students would prefer that the training involved a greater number of HCPs and to last longer. In addition, they emphasized that, in order to acquire adequate skills, the training methods should involve a practical session, a physical discussion with individuals who have undergone FGM/C who would share their experiences, and/or the use of standardized patients. For instance, 1 student stated,

*“This type of teaching might benefit greatly from the use of standardized patients” (Nursing student)*

## Discussion

We assessed the need and acceptability to train nursing and medical students in the sexual health care of patients with FGM/C in Tanzania. Findings indicated most participants were interested to very interested in receiving the training, found the topic acceptable, and had limited previous education in FGM/C care. Participants indicated the need to improve their competence and confidence in dealing with FGM/C, as well as mitigate the impact of FGM/C on the sexual, psychological, emotional, and overall health challenges experienced by women and girls who have undergone FGM/C. Despite not being asked about ending FGM/C, participants indicated that the training may have a role in ending FGM/C in their community.

### Need and acceptability

Our findings demonstrate the necessity and significance of developing a FGM/C curriculum to train frontline healthcare professionals to provide health care, including but not limited to sexual and reproductive healthcare. There was a clear gap between amount of training received and the identified need in this group of students in their final year of their programs. In addition, participants acknowledged the training to be relevant, and provided examples of FGM/C cases in their daily workflow. In Tanzania, studies show that there is a significant high rate of women of reproductive age who have undergone FGM/C and are still willing to practice FGM/C [8, 9]. FGM/C is widespread in Africa, including among women who migrate to Tanzania from other high prevalence areas, such as Sudan and Kenya [52]. To meet the current and future health needs from this population, HCPs need the competencies to diagnose, treat and manage complications among women/girls with FGM/C.

Previous research has demonstrated that FGM/C can have a negative impact on sexual function, though there is variability in the research that indicates that the type of FGM/C (i.e., the extent of the cutting) and socio-cultural factors play a role in the development of a sexual dysfunction [53–55]. HCPs in conservative countries, such as Tanzania, need training on how to broach the subject of sexual dysfunction and assess for existing sexual concerns, history of violence, and management of mistrust by patients [45, 48]. Sexual pain can be extreme for many women who have experienced FGM/C. HCPs can provide information on strategies for mitigating this pain and potentially improving sexual function [56, 57]. Yet it was unclear from our findings if the participants were aware of the benefits of sexual education or medical interventions, such as de-infibulation, on pain management.

### Perceived community benefits of the training

Health interventions targeted at women suffering from FGM/C-related complications can contribute to protect women and girls’ human rights from within the health system [2, 21]. The community awareness and mobilization on the harms of FGM/C in countries where the practice is concentrated is one of the proposed determinants in reducing its prevalence [58, 59]. The participants in our study were not prompted to reflect on larger community or societal needs, yet participants believed that increasing community awareness should be the first steps towards preventing and eliminating FGM/C. Participants recognized that training would equip them with the appropriate information to educate patients and the community about the effects of FGM/C, which could contribute to the abolition of the practice. These findings highlight the importance of raising community awareness while focusing on vulnerable populations, particularly in affected regions and pastoralist communities.

### Content and approaches to deliver the training

HCPs must know what to record in medical files, when to notify the authorities, and how to prevent the practice from being carried out [42]. Consistent with the literature, our study participants expressed a need for training objectives that focus on developing their skills in taking and documenting a proper medical history. Moreover, they recommended grounding the training on evidence-based education, using more simulations, and experiential exercises that focus on practical skills. These pedagogical methods could be effective in achieving measurable outcomes and improved patient care in clinical practice [60, 61]. On-site training through weekly seminars and interprofessional learning was mostly chosen as the preferred approach.

### Strengths, limitations, and future directions

We were able to survey over 400 students due to the existing research infrastructure at MUHAS, providing confidence in our findings that healthcare students in Tanzania are eager for further FGM/C training. This is the first step in developing a culturally tailored education-based intervention. However, there are several limitations that must be considered. This study has been limited to targeted health professionals; other stakeholders including patients, community and policy makers need to be involved for the training to reach its potential as an accepted approach to the caring of women/girls with FGM/C. In addition, this study was conducted only with students in Dar es Salaam city, Tanzania. This may limit the generalizability of the findings to HCPs (or patients) in other parts of Tanzania (rural regions). And we caution that the students in our sample may be eager for any extra training as compared to students who did not participate in the overall bigger study. This might inflate some estimates. The use of survey methods to obtain qualitative data limit the depth of that data. Despite these limitations, the study provides practical guidance for training nursing and medical students in Tanzania to care for women/girls who have experienced FGM/C. It adds to the existing literature that often focuses on training needs in the U.S. or Europe by assessing needs within Tanzania, allowing for the training to be tailored for an Afrocentric audience.

We recommend the development of training materials be grounded in the specific cultural needs of Tanzania. Future studies should involve in-depth interviews of students and other stakeholders to develop a more comprehensive understanding of the training needs, and attitudes towards FGM/C curriculum delivery within Tanzania. Training materials should address the skills needed to broach these challenging topics, including experiential teaching methods. Finally, globally there is a limited rigorous study of how FGM/C focused education impacts knowledge about and attitudes towards FGM/C. Following the development of suggested training materials, we recommend further study of the impact of the training.

## Conclusion

This study was designed to assess the need and acceptability of a training curriculum in sexual healthcare of women/girls with FGM/C targeting pre-service HCPs in Tanzania. Our findings underscore a high acceptability of the training, as a majority of participants were very interested to receive it. Participants viewed the training as most relevant and demonstrated a strong desire to become competent in dealing with FGM/C cases and participating in eradication initiatives. Designing a culturally sensitive curriculum and its delivery method that includes practical sessions with simulated patients, was considered the most beneficial and favorable. To improve quality of care to women who have undergone FGM/C, we recommend that those responsible for the training of medical, nursing and midwifery students should consider improving/adding the sexual healthcare to women/girls with FGM/C as a standard part of their education.

## Data Availability

The data analysed during the current study are available from the corresponding author upon reasonable request.

## Abbreviations

FGM/C: Female genital mutilation/cutting
HCP: Healthcare professionals
LMICs: Low- and Middle-income countries
MUHAS: Muhimbili University of Health and Allied Sciences
NIMR: National Institute for Medical Research
SD: Standard deviation
SDGs: Sustainable Development Goals
UNICEF: United Nations Children’s Fund
UNFPA: United Nations Population Fund
WHO: World Health Organization
